# Supplementary material for: Does attendance at a specialist antenatal clinic improve clinical outcomes in women with class III obesity compared with standard care? A retrospective case-note analysis
Source: BMJ Open. 2017 Jun 21;7(5):e015218. doi: 10.1136/bmjopen-2016-015218 (PMC5730005; doi:10.1136/bmjopen-2016-015218)
Supplement: Author's manuscript [file bmjopen-2016-015218.draft_revisions.pdf]

Attendance at a specialised antenatal clinic for obesity is associated with reduced rates of stillbirth and low birthweight and improved detection of gestational diabetes: a retrospective case-note analysis.

Journal:	*BMJ Open*
Manuscript ID	bmjopen-2016-015218
Article Type:	Research
Date Submitted by the Author:	16-Nov-2016
Complete List of Authors:	Denison, Fiona; The University of Edinburgh , MRC Centre for Reproductive Healht<br>MacGregor, Heather; University of Edinburgh Queen's Medical Research Institute<br>Stirrat, Laura; University of Edinburgh Queen's Medical Research Institute<br>Stevenson, Kerrie; University of Edinburgh Queen's Medical Research Institute<br>Norman, Jane; University of Edinburgh<br>Reynolds, Rebecca; University of Edinburgh,
**Primary Subject Heading**:	Obstetrics and gynaecology
Secondary Subject Heading:	Health services research
Keywords:	Obesity, Stillbirth, low birthweight

SCHOLARONE™ Manuscripts

For peer review only

Specialist clinic improves outcomes in maternal obesity

Attendance at a specialised antenatal clinic for obesity is associated with reduced rates of stillbirth and low birthweight and improved detection of gestational diabetes: a retrospective case-note analysis.

Denison FC[*], MacGregor H, Stirrat LI, Stevenson, KJ, Norman JE, Reynolds RM

Denison FC: Reader and Honorary Consultant in Maternal and Fetal Health, Tommy's Centre for Maternal and Fetal Health, University of Edinburgh MRC Centre for Reproductive Health, Queen's Medical Research Institute, 47 Little France Crescent, Edinburgh, EH16 4TY

MacGregor H: Medical Student, University of Edinburgh

Stirrat LI: Clinical Research Fellow, Tommy's Centre for Maternal and Fetal Health, University of Edinburgh MRC Centre for Reproductive Health, Queen's Medical Research Institute, 47 Little France Crescent, Edinburgh, EH16 4TY

Stevenson, KJ: Medical Student, University of Edinburgh

Norman JE: Professor of Maternal and Fetal Health, Tommy's Centre for Maternal and Fetal Health, University of Edinburgh MRC Centre for Reproductive Health, Queen's Medical Research Institute, 47 Little France Crescent, Edinburgh, EH16 4TY

Reynolds RM: Professor of Metabolic Medicine, British Heart Foundation Centre for Cardiovascular Science, Queen's Medical Research Institute, 47 Little France Crescent, Edinburgh, EH16 4TY

Corresponding author:

Dr Fiona Denison

Tommy's Centre for Maternal and Fetal Health

For peer review only

MRC Centre for Reproductive Health

Queen's Medical Research Institute

Edinburgh

0131 242 6449

Fiona.Denison@ed.ac.uk

For peer review only

Abstract

Objectives: To determine whether attendance at a specialised multidisciplinary antenatal clinic for women with Class III obesity (BMI>40kg/m^2) is associated with improved clinical outcomes compared to women receiving standard antenatal care

Design: Retrospective cohort study using routinely collected data from electronic patient record (TRAK)

Setting: Community or hospital based antenatal care

Participants: Women with a singleton pregnancy with Class III obesity who booked for antenatal care and delivered in one of two hospitals in NHS Lothian, Scotland, UK between 2008 – 2014. Maternal and offspring outcomes were compared in women who attended a specialised obesity clinic (attenders; n=511) compared to those who received standard antenatal care (non-attenders; n=502).

Main Outcome Measures: Outcomes including stillbirth, low birthweight, gestational diabetes, induction of labour and caesarean section.

Results: Compared to non-attenders, attenders were less likely to have a stillbirth (Odds Ratio (OR), 95% Confidence Interval (CI) 0.12, 0.06-0.97) and a low birthweight baby (OR, 95% CI 0.57, 0.33-0.99) and more likely to be screened for (100% vs 73.6%; <0.001) and diagnosed with (26.0% vs 12.5%; p<0.001) gestational diabetes, to require induction of labour (38.4% vs 29.9%, p=0.009), an elective (20.3% vs 17.7%; p<0.001) and emergency (23.9% vs 20.3%; p<0.001) caesarean section and attend antenatal triage one or more times during pregnancy (77.7% vs 53.1%; p<0.001). Attenders had a higher BMI (44.5% vs 43.2%; p<0.001) and were more likely to be nulliparous (46.0% vs 24.9%; p<0.001). There were no other differences in maternal demographic or maternal and offspring outcomes between attenders and non-attenders.

For peer review only

Conclusions: Attendance at a specialised antenatal clinic for obesity is associated with reduced rates of stillbirth and low birthweight and improved detection of gestational diabetes. The improvement in clinical outcomes is associated with an increase in healthcare attendance to obstetric triage and clinical interventions including induction of labour and caesarean section.

Keywords

Obesity, stillbirth, low birthweight

For peer review only

Specialist clinic improves outcomes in maternal obesity

Article Summary - Strengths and limitations of this study

- This study compares maternal and offspring outcomes in women with Class III obesity who attend a specialist obesity antenatal clinic compared to those who do not.
- A strength of our study is that we were able to compare important clinical outcomes in women and offspring such as stillbirth and low birthweight.
- The use of routinely collected clinical data means that our results are relevant to clinical practice in which multiple different care pathways exist.
- The stillbirth findings and causality need to be interpreted with caution due to the small sample size and attenuation of findings in adjusted analyses.
- As a retrospective cohort study using routinely collected data from electronic patient record, results must be interpreted with caution because of potential bias from confounding.

For peer review only

Introduction

Maternal obesity is the most common co-morbidity of pregnancy. In the UK, approximately 20% of pregnant women are obese and 2% have very severe obesity (Class III obesity, body mass index BMI ≥ 40 kg/m^2) [1]. Maternal obesity is associated with increased risks for adverse maternal and offspring health including gestational diabetes mellitus (GDM), thromboembolic and hypertensive complications, caesarean section, macrosomia and stillbirth [2-5]. Managing these complications has significant cost implications for delivery of antenatal care [2, 4] [6].

There is recognition that obstetric management of the obese should be consultant led and involve a multi-disciplinary team to improve outcome [7, 8]. These recommendations are embedded in clinical guidelines and standards of care produced by a number of countries [8-13]. However, there is a paucity of evidence demonstrating that multidisciplinary care and adherence to guidelines results in improved maternal and offspring outcomes in maternal obesity. There is also less consensus about how multidisciplinary care should be delivered, and a concern that in areas of high obesity prevalence specialist obesity clinics are unlikely to be feasible due to cost and the numbers of women who would potentially need to be seen [13].

Women with Class III obesity are at particularly high risk of adverse maternal and offspring outcome [14]. In 2008 we therefore set up a specialist antenatal clinic for women with Class III obesity living in Edinburgh and the surrounding Lothian area with the aim of improving maternal and offspring outcomes. At their first antenatal appointment, which is generally prior to 12 weeks gestation, women with a BMI >40kg/m^2 are offered referral to the specialist clinic or can choose to continue to receive standard antenatal care. We have a pan-Lothian guideline for clinical management of pregnancies in women with obesity (Class I, II and III) so that the same care pathway is offered, regardless of who or where it is delivered. All women with Class III obesity should therefore receive the same standard of care. We hypothesised that maternal and offspring outcomes would be better in women who had their antenatal care provided by a multidisciplinary specialist clinic as opposed to receiving standard antenatal care. To test this hypothesis, we undertook a retrospective case-note review of all women with a BMI>40kg/m^2

For peer review only - http://bmjopen.bmj.com/site/about/guidelines.xhtml

who delivered in Lothian between 2008 and 2014 and compared clinical outcomes in women who attended for specialist antenatal care compared to those who received standard antenatal care.

Methods

Study population

We performed a retrospective case-note review of all women with Class III obesity with a singleton pregnancy who booked for antenatal care and delivered in either of two hospitals in NHS Lothian trust between 2008 and 2014. The Simpson Centre for Reproductive Health at the Royal Infirmary of Edinburgh is a tertiary referral centre with more than 6,500 deliveries per annum. St John's Hospital, Livingston, is a district general hospital with approximately 2,600 deliveries per annum. Women were excluded if they had not delivered by the end of December 2014, had a multiple pregnancy (n=28), or booked later than 20 weeks gestation (n=18) because this meant they would have missed the gestational window for early screening for GDM [15]. This study was an approved by our local audit committee.

Clinical care pathway

Women attending the specialist clinic at the Simpson Centre for Reproductive Health, Royal Infirmary of Edinburgh receive multidisciplinary consultant-led care throughout pregnancy from obstetricians, specialist midwives, diabetologists, anaesthetists, dieticians and other specialists as clinically indicated. At their first appointment (~10-16 weeks gestation), women are reviewed individually by a dietician with specialist expertise in weight management during pregnancy and given tailored advice about healthy eating and weight management during pregnancy. They are advised to have early screening for GDM with a fasting blood glucose between 12-16 weeks and late screening using a 75g oral glucose tolerance test between 24-28 weeks, as per the Scottish Intercollegiate Guidelines [15]. If a woman has pre-existing Type 2 diabetes or is diagnosed with GDM during pregnancy, her care remains within the specialist clinic. At each visit, women are weighed, counselled about the maternal and offspring risks associated

For peer review only - http://bmjopen.bmj.com/site/about/guidelines.xhtml

with maternal obesity, and their blood pressure is measured with appropriate sized cuffs. Women are commenced on 75mg aspirin if they have additional risk factors for pre-eclampsia such as a blood pressure of >140/90 mmHg at antenatal booking or primiparity as per national guidelines [16]. All women have postnatal thromboprophylaxis with low molecular weight heparin, with antenatal thromboprophylaxis being commenced if additional risk factors develop [16]. Fetal growth is monitored by serial growth scans at 28, 32 and 36 weeks. All women receive a personalised delivery plan and an anaesthetic review in the third trimester to discuss intrapartum pain management with specific consideration given to obesity related co-morbidities with implications for analgesia and anaesthesia.

Women who do not attend the specialist clinic receive guideline based consultant led care in hospital or community based antenatal clinics. The main difference in care between women who attend the specialist clinic and those who do not, is that if non-attenders develop a complication such as gestational diabetes or needs anaesthetic review, they need to attend a separate specialist clinic to receive this additional care. For women who attend the obesity clinic, this care is centralised in a single multi-professional clinic.

To compare maternal and offspring outcomes by antenatal care setting, women were categorised as 'attenders' if they attended for two or more appointments at the specialist clinic with the first appointment being before 20 weeks. The rationale for this was that such women would have received early dietary advice and counselling about the importance of attending for early screening for GDM.

Data Collection

Maternal and offspring data were acquired from the maternity electronic patient records database TRAK (supplied by Intersystems), clinical biochemistry database APEX (ApexHealthware) and the neonatal unit electronic patient records database BadgerNet (supplied by Clevermed) systems.

The following data were collected from the maternal record at booking: maternal age, BMI (kg/m^2), ethnicity (white, other), parity (P0, P1, P2 or more), smoking status (current, former, never), deprivation

For peer review only

quintile (a postcode based Scottish Index of Multiple Deprivation from 2012 with five groups ranging from most deprived index (1) to least deprived index (5))[17] and systolic and diastolic blood pressure (mmHg).

Maternal outcomes collected were hypertension (pre-existing, gestational, pre-eclampsia), diabetes (pre-existing, GDM), onset of labour (no labour, spontaneous onset, induced), delivery method (elective caesarean, emergency caesarean, instrumental, spontaneous vaginal), blood loss at delivery and antenatal obstetric attendances. The prevalence of GDM was determined according to (i) the rates of GDM from diagnoses entered into the electronic patient record and (ii) evaluating whether blood glucose values found on the electronic databases conferred a diagnosis of GDM. Diagnostic accuracy of GDM was determined according to Scottish Intercollegiate Guidelines which utilised the World Health Organisation (WHO) recommended thresholds [18] until March 2010 when updated thresholds were published based on the International Association of the Diabetes and Pregnancy Study Groups [19].

Offspring outcomes collected were gender, birthweight, birthweight centile [20], macrosomia (defined as a birthweight ≥4000g), low birthweight (defined as a birthweight ≤ 2500g), gestation of delivery, preterm birth (defined as birth <259 days gestation) and outcome (livebirth, stillbirth).

All data were anonymised with personal identifiers removed before analysis. To maximize accuracy and to minimise missing data all records were reviewed by HM and LS, glucose data was reviewed by KS and LS with any discrepancies reviewed by FD, RR. For stillbirths, a perinatal pathologist examined placental pathology as is routine clinical practice. HM and LS independently identified risk factors and categorised the likely causality of the stillbirths. Stillbirth causation was checked and verified by a third investigator (FD). All investigators were blinded to whether a woman did or did not attend the specialist clinic until risk factors and likely causality was agreed for all stillbirths.

Statistical Analysis

For peer review only

For peer review only - http://bmjopen.bmj.com/site/about/guidelines.xhtml

Data were analysed using the Statistical Package for the Social Sciences (SPSS) Version 21. Differences in the characteristics and clinical outcomes between the women who attended the specialist obesity clinic and those who received standard care were tested using the student's t-test if the variable was continuous or the chi-squared test for categorical variables. Logistic regression was used to adjust for BMI and parity. A p-value <*0.05* was considered statistically significant.

Results

Demographics

Maternal demographics are demonstrated in Table 1. Compared to non-attenders, women who attended the specialist clinic had a higher BMI, and were more likely to be primiparous. There were no differences in age, ethnicity, smoking status, deprivation quintile and systolic or diastolic blood pressure at booking between attenders and non-attenders.

Maternal outcomes

Maternal outcomes are demonstrated in Table 2. After excluding women with pre-existing Type 1 and Type 2 diabetes, all women who attended the specialist clinic had a screening test with sufficient information being collected to confirm or exclude a diagnosis of GDM. In contrast, 26.4% (128/484) of non-attenders either had no screening test for GDM or insufficient information was collected for a diagnosis of GDM to be made. The clinical diagnosis of GDM from the patient record matched the diagnosis from blood glucose levels in all women who attended the specialist clinic. In contrast, in non-attenders, when the notes and actual blood glucose values were compared, the 'wrong' diagnosis was made in 17 women. One woman was incorrectly diagnosed with GDM when her screening test for GDM was normal. A further 16 woman had a positive diagnostic test for GDM according to glucose values obtained during a glucose tolerance test but the diagnosis was missed and these women were incorrectly labelled as not having GDM (and did not therefore receive treatment).

For peer review only - http://bmjopen.bmj.com/site/about/guidelines.xhtml

Specialist clinic improves outcomes in maternal obesity

Compared to non-attenders, women who attended the specialist clinic were more likely to have their labour induced, to have a caesarean or instrumental vaginal delivery. Specialist clinic attenders had a higher blood loss at delivery than non-attenders even after adjusting for mode of delivery, BMI, age and parity (p=0.02). They were also more likely to attend obstetric triage one or more times during pregnancy. Rates of pre-existing chronic hypertension and hypertensive complications (gestational hypertension and pre-eclampsia) were low in both attenders and non-attenders. Rates of Type 2 diabetes were higher in non-attenders compared to attenders.

Offspring outcomes

The clinical details for the offspring outcomes are demonstrated in Table 3. Compared to non-attenders, attenders were less likely to have a stillbirth (Odds Ratio (OR), 95% Confidence Interval (CI) 0.12, 0.06-0.97) and a low birthweight baby (OR, 95% CI 0.57, 0.33-0.99). The lower stillbirth outcomes in attenders were attenuated in analyses adjusting for BMI and parity (adjusted OR (AOR), 95% CI 0.14, 0.02-1.17) but the lower risk of having a low birthweight baby (in the attendees) was strengthened in adjusted analyses (AOR, 95% CI 0.52, 0.29-0.93). The clinical details of the women who had a stillbirth are demonstrated in Table 4. In non-attenders, an additional risk factor for stillbirth was identified in 7 women and a probable cause for stillbirth was identified in all 8 women. No additional risk factors or cause was identified in the one woman who had a stillbirth who attended the specialist clinic.

Discussion

In this retrospective case-note review, we demonstrated that women with Class III obesity who attended a specialist multidisciplinary antenatal clinic were less likely to have a stillbirth and low birthweight infant and more likely to be tested, correctly diagnosed with and treated for GDM, and to have an induction of labour, caesarean section and higher blood loss at delivery compared to those receiving standard antenatal care. These differences in outcomes were accompanied by increased attendance at obstetric triage.

Main Findings

For peer review only - http://bmjopen.bmj.com/site/about/guidelines.xhtml

Specialist clinic improves outcomes in maternal obesity

A key study finding was that rates of stillbirth and low birthweight were lower in women who attended the clinic compared to those who did. Compared to non-attenders, women who attended the specialist clinic had a higher BMI, and were more likely to be primiparous. Given that primiparity and higher BMI are independently associated with increased risk of stillbirth and low birthweight [21-23], we expected that rates of stillbirth and low birthweight would be higher in attenders compared to non-attenders. However, we found the converse to be the case, with fewer stillborn and low birthweight babies being born to attenders, even after adjusting for parity and BMI.

The stillbirth rate in women who attended the specialist clinic was 2 per 1000 compared to a rate of 7 per 1000 for women with a BMI>40kg/m^2 who delivered in Scotland in 2011 - 2012 [24]. To validate this finding, three investigators who were blinded to whether women did or did not attend the clinic independently checked the stillbirth data. It was striking that additional risk factors were identified in 7 and a cause for stillbirth identified in all 8 non-attenders who had a stillbirth but no additional risk factors or cause was identified in the one woman who had a stillbirth who did attend the clinic. We accept that rates of unexplained stillbirth are generally reported as being 20 – 25% which is much higher than what we found in our study. We therefore acknowledge the stillbirth findings and causality need to be interpreted with caution due to the small sample size and attenuation of findings in adjusted analyses. However it is tempting to speculate that the continuity of care together with the education of women by the multidisciplinary clinic team raised increased awareness of the importance of risk factors such as reduced fetal movements amongst attenders and this may have led to them presenting earlier and being delivered prior to stillbirth occurring. Future studies such as the AFFIRM clinical trial (NCTT01777022, due to complete in 2017) are designed to address this in the general antenatal population.

Strengths and Limitations

A strength of our study is that we were able to compare important clinical outcomes in women and offspring such as stillbirth. We also used routinely collected clinical data meaning that our results are relevant to clinical practice in which multiple different care pathways exist. We accept that a limitation of

For peer review only - http://bmjopen.bmj.com/site/about/guidelines.xhtml

our study is that this was a retrospective case-note review and our sample size was therefore limited by the study population. For the majority of data fields, other than smoking status (43.8% missing), there was a relatively low proportion of missing data. For the smoking variable, this was due to smoking status not being a mandatory field for recording on the electronic clinical record prior to 2012. The study was also not randomised, so women could choose whether to attend the specialist clinic. However, apart from differences in maternal BMI and primiparity between attenders and non-attenders, all other demographic factors were comparable between groups. Given that the clinical outcomes were better in women attending the specialist clinic who were arguably at higher risk than the non-attenders due to their higher BMI and more likely to be primiparous, we believe that our finding that multidisciplinary care improves clinical outcomes in pregnant women with Class III obesity compared to standard care is clinically important.

Interpretation

In other general and high-risk populations, pregnancy outcomes tend to be worse in women who either under- or do not attend for any antenatal care [25]. However, although we categorised women into attenders or non-attenders, this was only in relation to whether they attended the specialist clinic for antenatal care. In 1993, the landmark Changing Childbirth Report [26], which was built on the 1992 Winterton Report, reversed the official policy that hospital is always the safest place for birth and emphasised the importance of maternal choice, control and continuity of carer for women. These recommendations, which were made over 20 years ago are still as relevant today, and frame the rhetoric and delivery of antenatal care across the UK [27-30]. In Lothian, all women receiving community-led care have a named midwife who coordinates their care. This midwife is part of a community team which has a defined case-load. This model ensures that there is continuity of care for a woman at both the individual midwife and midwifery team-level. If a woman is deemed high risk (such as would be the case in women with Class III obesity), she is also designated a named Consultant to oversee her care. Despite this model of continuity of care, our study demonstrates that maternal and offspring outcomes are better

For peer review only

in women who attend a hospital based specialist clinic compared to those who receive standard antenatal care.

Although specialist clinics have been advocated as a way of improving maternal and offspring outcomes, there is currently a paucity of evidence from randomised controlled trials about the benefits and harms of 'specialist' antenatal clinics compared with 'standard' antenatal care for women [31]. For example, systematic reviews of randomised controlled trials have concluded that there is currently limited information to assess the role of 'specialist' antenatal clinics for women with a multiple pregnancy [32] and no clear evidence that 'specialist' clinics reduce the number of preterm births [33]. Given that the antenatal care pathway followed was the same in attenders compared to non-attenders, it is not clear why maternal and offspring outcomes were better in women who attended the specialist clinic compared to those who did not. A recent systematic review by Sandall et al. highlighted the importance of continuity of care, demonstrating that pregnant women receiving midwife-led continuity models of care had at least comparable clinical outcomes and were likely to experience less intervention [34]. It is therefore plausible that the continuity of care that the specialist multidisciplinary team provided enabled compromised pregnancies to be identified more accurately and interventions such as induction of labour to be targeted more appropriately compared to those women receiving standard care.

Conclusion

In summary, our study demonstrates that attendance at a multidisciplinary specialist antenatal clinic improves maternal and offspring outcomes in women with Class III obesity. This challenges current recommendations that women with very Class III obesity can be effectively managed outside a specialist service. Further research is needed to identify the most appropriate and economic model of care for women with Class III obesity to optimise maternal and offspring outcomes.

Acknowledgements

For peer review only

We would like to acknowledge Mr Allyn Dick for assistance in extracting the clinical data from maternity TRAK.

Copyright

The Corresponding Author has the right to grant on behalf of all authors and does grant on behalf of all authors, a worldwide licence to the Publishers and its licensees in perpetuity, in all forms, formats and media (whether known now or created in the future), to i) publish, reproduce, distribute, display and store the Contribution, ii) translate the Contribution into other languages, create adaptations, reprints, include within collections and create summaries, extracts and/or, abstracts of the Contribution, iii) create any other derivative work(s) based on the Contribution, iv) to exploit all subsidiary rights in the Contribution, v) the inclusion of electronic links from the Contribution to third party material where-ever it may be located; and, vi) licence any third party to do any or all of the above.

Disclosure of Interests

The authors have no interests to disclose.

Contribution to authorship

FD and RR conceived the study and drafted the paper. FD, HM, LS, KS, JN and RR designed the study. FD, HM, LS, KS and RR acquired and analysed the data. All authors interpreted the data, revised the paper critically for important intellectual content and approved the final version.

Details of Ethics Approval

This study was an approved by our local audit committee.

Funding

We acknowledge funding from Tommy's the Baby Charity

For peer review only

References

1. Report, C.f.M.a.C.E., *Maternal obesity in the UK: findings from a national project*, in *Centre for Maternal and Child Enquiries*2010: Chiltern Court, 188 Baker Street, London, NW1 5SD.
2. Denison, F.C., et al., *Association between maternal body mass index during pregnancy, short-term morbidity, and increased health service costs: a population-based study.* BJOG, 2014. **121**(1): p. 72-81; discussion 82.
3. Denison, F.C. and C. Chiswick, *Improving pregnancy outcome in obese women.* Proc Nutr Soc, 2011. **70**(4): p. 457-64.
4. Denison, F.C., et al., *Increased Maternal BMI is associated with an Increased Risk of Minor Complications during Pregnancy With Consequent Cost Implications.* BJOG, 2009. **116**(11): p. 1467-72.
5. Reynolds, R.M., et al., *Maternal and Offspring Outcomes of Pregnancies associated with Severe Obesity: Study design and preliminary data from the Hormones and Inflammation in Obese Pregnancy Study.* J of Developmental Origins of Health and Disease 2009: p. P7A-318.
6. Morgan, K.L., et al., *Obesity in pregnancy: a retrospective prevalence-based study on health service utilisation and costs on the NHS.* BMJ Open, 2014. **4**(2): p. e003983.
7. Ghaffari, N., S.K. Srinivas, and C.P. Durnwald, *The multidisciplinary approach to the care of the obese parturient.* Am J Obstet Gynecol, 2015. **213**(3): p. 318-25.
8. Jarvie, E. and J.E. Ramsay, *Obstetric management of obesity in pregnancy.* Semin Fetal Neonatal Med, 2010. **15**(2): p. 83-8.
9. *ACOG Committee Opinion No. 650: Physical Activity and Exercise During Pregnancy and the Postpartum Period.* Obstet Gynecol, 2015. **126**(6): p. e135-42.
10. *ACOG Practice Bulletin No 156: Obesity in Pregnancy.* Obstet Gynecol, 2015. **126**(6): p. e112-26.
11. Alavi, N., et al., *Comparison of national gestational weight gain guidelines and energy intake recommendations.* Obes Rev, 2013. **14**(1): p. 68-85.
12. Fitzsimons, K.J. and J. Modder, *Setting maternity care standards for women with obesity in pregnancy.* Semin Fetal Neonatal Med, 2010. **15**(2): p. 100-7.
13. RCOG, *Management of Women with Obesity in Pregnancy* in *RCOG* CMACE/RCOG, Editor 2010, CMACE/RCOG: London.
14. Lutsiv, O., et al., *The effects of morbid obesity on maternal and neonatal health outcomes: a systematic review and meta-analyses.* Obes Rev, 2015. **16**(7): p. 531-46.
15. Network, S.I.G., *Management of Diabetes: A national clinical guideline*, 2014, Scottish Intercollegiate Guidelines Network: Edinburgh.
16. NICE, *Hypertension in pregnancy: diagnosis and management: CG107*, 2011.
17. Government, T.S., *Scottish Index of Multiple Deprivation 2012. A National Statistics Publication for Scotland*, in *National Statistics*2012, The Scottish Government.
18. (SIGN), S.I.G.N., *Management of Diabetes. SIGN publication no.55*, 2001, SIGN: Edinburgh.
19. International Association of, D., et al., *International association of diabetes and pregnancy study groups recommendations on the diagnosis and classification of hyperglycemia in pregnancy.* Diabetes Care, 2010. **33**(3): p. 676-82.
20. Bonellie, S., et al., *Centile charts for birthweight for gestational age for Scottish singleton births.* BMC Pregnancy Childbirth, 2008. **8**: p. 5.
21. Aune, D., et al., *Maternal body mass index and the risk of fetal death, stillbirth, and infant death: a systematic review and meta-analysis.* JAMA, 2014. **311**(15): p. 1536-46.
22. Flenady, V., et al., *Stillbirths: the way forward in high-income countries.* Lancet, 2011. **377**(9778): p. 1703-17.
23. Shah, P.S. and L.B.W.P.T.b. Knowledge Synthesis Group on Determinants of, *Parity and low birth weight and preterm birth: a systematic review and meta-analyses.* Acta Obstet Gynecol Scand, 2010. **89**(7): p. 862-75.
24. ISD. *Stillbirth and Infant Deaths*. 2016 [cited 2016 14th November, 2016]; Available from: http://www.isdscotland.org/health-Topics/Maternity-and-Births/Stillbirth-and-Infant-Deaths/.

For peer review only

For peer review only - http://bmjopen.bmj.com/site/about/guidelines.xhtml

Specialist clinic improves outcomes in maternal obesity

25. Raatikainen, K., N. Heiskanen, and S. Heinonen, *Under-attending free antenatal care is associated with adverse pregnancy outcomes.* BMC Public Health, 2007. **7**: p. 268.
26. Health, D.o., *Changing childbirth. Part 1: report of the Expert Maternity Group.*, D.o. Health, Editor 1993, Department of Health: London.
27. Health, D.o., *National Service Framework for children, young people and maternity services: maternity services*, 2004, Department of Health: London.
28. Health, D.o., *Matnerity matters: choice, access and continuity of care in a safe service*, 2007, Department of Health: London.
29. Government, S. *A Refreshed Framework for Maternity Care in Scotland*. 2011 [cited 2016 24th October, 2016]; Available from: http://www.gov.scot/resource/doc/337644/0110854.pdf.
30. *Better Births - Improving outcomes of maternity services in England*, N.M. Review, Editor 2016.
31. Dodd, J.M. and C.A. Crowther, *Specialised antenatal clinics for women with a multiple pregnancy for improving maternal and infant outcomes.* Cochrane Database Syst Rev, 2012(8): p. CD005300.
32. Dodd, J.M., T. Dowswell, and C.A. Crowther, *Specialised antenatal clinics for women with a multiple pregnancy for improving maternal and infant outcomes.* Cochrane Database Syst Rev, 2015(11): p. CD005300.
33. Whitworth, M., et al., *Specialised antenatal clinics for women with a pregnancy at high risk of preterm birth (excluding multiple pregnancy) to improve maternal and infant outcomes.* Cochrane Database Syst Rev, 2011(9): p. CD006760.
34. Sandall, J., et al., *Midwife-led continuity models versus other models of care for childbearing women.* Cochrane Database Syst Rev, 2016. **4**: p. CD004667.

For peer review only

For peer review only - http://bmjopen.bmj.com/site/about/guidelines.xhtml

Table 1: Demographics of population

	Attenders (n=511)	Non-attenders (n=502)	P value
Age (years; mean (SD))	29.8 (5.4)	29.3 (5.5)	0.11
BMI (kg/m^2; mean (SD))	44.5 (4.3)	43.2 (3.1)	<0.001
Ethnicity (n (%))*			0.35
White	441 (94.6%)	432 (92.9%)	
Other	25 (5.4%)	33 (7.1%)	
Parity (n (%))			<0.001
0	235 (46.0%)	125 (24.9%)	
1	161 (31.5%)	212 (42.2%)	
2 or more	115 (22.5%)	165 (32.9%)	
Smoking status (n (%))*			0.51
Current	45 (17.2%)	42 (13.7%)	
Former	63 (24.0%)	79 (25.7%)	
Never	154 (58.8%)	186 (60.6%)	
Deprivation quintile (n (%))[1]*			0.07
1	140 (27.7%)	108 (22.2%)	
2	141 (27.9%)	150 (30.9%)	
3	95 (18.8%)	107 (22.0%)	
4	66 (13.1%)	74 (15.2%)	
5	63 (12.5%)	47 (9.7%)	
Systolic blood pressure (mmHg; mean (SD))	122 (11.9)	122 (11.1)	0.79
Diastolic blood pressure (mmHg)[1]	75 (9.0)	75 (8.0)	0.98

*missing data includes n=82 (8%) from ethnicity, n=444 (44%) from smoking and n=12 (1.2%) from deprivation quintile. Missing data is high from smoking as this was not a mandatory field on the electronic record until 2012; [1]deprivation quintile where 1 is the most and 5 the least deprived.

For peer review only

436 **Table 2: Maternal outcomes**

437

	Attenders N=511	Non-attenders N=502	*P* value
Pre-existing co-morbidities			
Type 2 diabetes (n; %)	2 (0.4%)	12 (2.4%)	0.008
Hypertensive complications			0.27
Chronic hypertension (n; %)	16 (1.6%)	11 (1.1%)	
Gestational hypertension (n; %)	18 (1.8%)	16 (1.6%)	
Pre-eclampsia (n; %)	31 (3.1%)	25 (2.5%)	
Gestational diabetes*			
Screening/diagnostic test performed (n; %)	496 (100%)	356 (73.6%)	<0.001
Prevalence (n; %)	129 (26.0%)	61 (12.5%)	<0.001
Labour and delivery			
Onset labour (n; %)			0.009
No labour	111 (21.7%)	109 (21.7%)	
Spontaneous onset	204 (39.9%)	243 (48.4%)	
Induction	196 (38.4%)	150 (29.9%)	
Delivery method (n; %)			
Elective caesarean	103 (20.2%)	89 (17.7%)	<0.001
Emergency caesarean	122 (23.9%)	102 (20.3%)	
Instrumental	56 (11.0%)	23 (4.6%)	
Spontaneous vertex	229 (44.9%)	288 (57.4%)	
Blood loss at delivery (mls; mean (SD))	575 (464)	465 (387)	<0.001
Obstetric triage attendances (n; %)			<0.001
0	108 (21.1%)	229 (45.6%)	
1	132 (25.8%)	104 (20.7%)	
2	93 (18.2%)	70 (13.9%)	
3 or more	172 (33.7%)	93 (18.5%)	

438 *Denominator excludes women with pre-existing diabetes (type 1 or 2) or those who were not managed at the
439 tertiary referral centre. In attenders and non-attenders, the prevalence is based on blood glucose levels and not the
440 clinical diagnosis recorded in the notes.

441

442

443

444

445

For peer review only

446 **Table 3: Offspring outcomes**

447

	Attenders N=511	Non-attenders N=502	Significance (*P* value)
Gender (n; %)			0.34
Female	238 (46.6%)	249 (49.6%)	
Male	273 (53.4%)	253 (50.4%)	
Birthweight (g; mean (SD))	3576 (635)	3559 (664)	0.69
Macrosomia[1] (n; %)	31 (6.1%)	26 (5.2%)	0.54
Low birthweight[2] (n; %)	21 (4.1%)	35 (7.0%)	0.04
Gestation (days; mean (SD))	277 (14.1)	277 (14.7)	0.82
Preterm birth[3] (n; %)	40 (7.8%)	39 (8.4%)	0.97
Outcome (n; %)			
Livebirth	510 (99.8%)	494 (98.4%)	0.02
Stillbirth	1 (0.2%)	8 (1.6%)	

448 [1]macrosomia defined as birthweight of 4000g or more; [2]low birthweight defined as birthweight of 2500g or lower;
449 [3]preterm birth defined as birth before 259 days gestation.

450

451

For peer review only

452
453
454 **Table 4: Details of stillbirths**
455

Case	Demographics			Risk factors	Outcome	Birthweight centile[1]	Cause
	Age (years)	Parity	BMI (kg/m^2)				
NA1	31	P2	42	Smoker, type 2 diabetes, RFM	33+5 weeks, male, 2050g	25th – 50th	Uncontrolled hypertension, abruption
NA2	32	P1	42	No risk factors	30+5 weeks, female, 700g,	<3rd	IUGR, placental insufficiency
NA3	38	P4	42	RFM	37 weeks, male, 2720g,	10th – 25th	Severe pre-eclampsia, abruption
NA4	32	P2	45	Smoker, RFM	36 weeks, male, 2160g,	5th – 10th	Acute intra-uterine hypoxia
NA5	26	P2	47	Smoker, RFM, isolated congenital anomaly	35+5 weeks, female, 2155g,	10th – 25th	Congenital anomaly
NA6	32	P2	52	Smoker	30+5 weeks, female, 1620g,	75th – 90th	Abruption
NA7	27	P2	40	Type 2 diabetes, RFM	38+2 weeks, male, 3370g,	50th – 75th	Poorly controlled diabetes
NA8	21	P0	40	Smoker	26+3 weeks, female, 750g,	25th – 50th	IUGR, placental insufficiency
A1	20	P1	41	No risk factors	39+5 weeks, male, 3725g,	50th – 75th	Unexplained

456 Key: NA non-attender, A attender, [1] birthweight centile defined by (Bonellie et al., 2008); BMI body mass index,
457 RFM reduced fetal movements, IUGR intrauterine growth restriction, placental insufficiency diagnosed by
458 perinatal pathologist
459

For peer review only

The RECORD statement – checklist of items, extended from the STROBE statement, that should be reported in observational studies using routinely collected health data.

	Item No.	STROBE items	Location in manuscript where items are reported	RECORD items	Location in manuscript where items are reported
**Title and abstract**					
	1	(a) Indicate the study's design with a commonly used term in the title or the abstract (b) Provide in the abstract an informative and balanced summary of what was done and what was found	(a) Page 1<br>(b) Page 4-5	RECORD 1.1: The type of data used should be specified in the title or abstract. When possible, the name of the databases used should be included.<br><br>RECORD 1.2: If applicable, the geographic region and timeframe within which the study took place should be reported in the title or abstract.<br><br>RECORD 1.3: If linkage between databases was conducted for the study, this should be clearly stated in the title or abstract.	1.1: Lines 52-53<br><br>1.2: Lines 57-58<br><br>1.3: Not applicable
**Introduction**					
Background rationale	2	Explain the scientific background and rationale for the investigation being reported	Page 6 &7, lines 83 - 111		
Objectives	3	State specific objectives, including any prespecified hypotheses	Page 6 & 7, lines 106 - 111		
**Methods**					
Study Design	4	Present key elements of study design early in the paper	Page 7 & 8, lines 117 - 155		
Setting	5	Describe the setting, locations, and relevant dates, including periods of recruitment, exposure, follow-up, and data collection	Page 7, lines 117 - 124		
Participants	6	*(a) Cohort study* - Give the eligibility criteria, and the	(a) Page 7 & 8, lines 127 – 155	RECORD 6.1: The methods of study population selection (such as codes or	6.1: Lines 152 – 155

For peer review only

		sources and methods of selection of participants. Describe methods of follow-up<br>*Case-control study* - Give the eligibility criteria, and the sources and methods of case ascertainment and control selection. Give the rationale for the choice of cases and controls<br>*Cross-sectional study* - Give the eligibility criteria, and the sources and methods of selection of participants<br><br>*(b) Cohort study* - For matched studies, give matching criteria and number of exposed and unexposed<br>*Case-control study* - For matched studies, give matching criteria and the number of controls per case	 <br><br><br><br><br><br><br><br><br><br><br><br>(b) Page 8, lines 152 - 155	algorithms used to identify subjects) should be listed in detail. If this is not possible, an explanation should be provided.<br><br>RECORD 6.2: Any validation studies of the codes or algorithms used to select the population should be referenced. If validation was conducted for this study and not published elsewhere, detailed methods and results should be provided.<br><br>RECORD 6.3: If the study involved linkage of databases, consider use of a flow diagram or other graphical display to demonstrate the data linkage process, including the number of individuals with linked data at each stage.	 <br><br><br><br>6.2: Not applicable<br><br><br><br><br><br>6.3: Not applicable
Variables	7	Clearly define all outcomes, exposures, predictors, potential confounders, and effect modifiers. Give diagnostic criteria, if applicable.	Page 8 & 9: lines 158 - 189	RECORD 7.1: A complete list of codes and algorithms used to classify exposures, outcomes, confounders, and effect modifiers should be provided. If these cannot be reported, an explanation should be provided.	Page 8 & 9: lines 158 - 189
Data sources/ measurement	8	For each variable of interest, give sources of data and details of methods of assessment (measurement).<br>Describe comparability of assessment methods if there is more than one group	Page 8 & 9: lines 158 - 189		
Bias	9	Describe any efforts to address potential sources of bias	Page 9: lines 184 - 189		
Study size	10	Explain how the study size was	Page 7: lines 117 -		

For peer review only

		arrived at	121		
Quantitative variables	11	Explain how quantitative variables were handled in the analyses. If applicable, describe which groupings were chosen, and why	Page 8 & 9: lines 158 - 189		
Statistical methods	12	(a) Describe all statistical methods, including those used to control for confounding<br>(b) Describe any methods used to examine subgroups and interactions<br>(c) Explain how missing data were addressed<br>(d) *Cohort study* - If applicable, explain how loss to follow-up was addressed<br>*Case-control study* - If applicable, explain how matching of cases and controls was addressed<br>*Cross-sectional study* - If applicable, describe analytical methods taking account of sampling strategy<br>(e) Describe any sensitivity analyses	(a) – (e) Page 10: lines 191 - 196		
Data access and cleaning methods		..		RECORD 12.1: Authors should describe the extent to which the investigators had access to the database population used to create the study population.<br><br>RECORD 12.2: Authors should provide information on the data cleaning methods used in the study.	12.1: Page 9, lines 184 – 189<br><br>12.2: Page 9, lines 184 - 189
Linkage		..		RECORD 12.3: State whether the study included person-level, institutional-	12.3: Not applicable

For peer review only

				level, or other data linkage across two or more databases. The methods of linkage and methods of linkage quality evaluation should be provided.	
**Results**					
Participants	13	(a) Report the numbers of individuals at each stage of the study (*e.g.*, numbers potentially eligible, examined for eligibility, confirmed eligible, included in the study, completing follow-up, and analysed)<br>(b) Give reasons for non-participation at each stage.<br>(c) Consider use of a flow diagram	(a - b) Page 6: lines 121 – 124 and page 10: lines 200<br>(c) Not applicable	RECORD 13.1: Describe in detail the selection of the persons included in the study (*i.e.,* study population selection) including filtering based on data quality, data availability and linkage. The selection of included persons can be described in the text and/or by means of the study flow diagram.	13.1: Page 6: lines 121 – 124 and page 10: lines 200
Descriptive data	14	(a) Give characteristics of study participants (*e.g.*, demographic, clinical, social) and information on exposures and potential confounders<br>(b) Indicate the number of participants with missing data for each variable of interest<br>(c) *Cohort study* - summarise follow-up time (*e.g.*, average and total amount)	(a) Page 9: lines 199 – 203<br><br>(b) Table 1<br><br>(c) Not applicable		
Outcome data	15	*Cohort study* - Report numbers of outcome events or summary measures over time<br>*Case-control study* - Report numbers in each exposure category, or summary measures of exposure<br>*Cross-sectional study* - Report numbers of outcome events or summary measures	Page 9 & 10, lines 205 – 235 and Tables 1 - 4		
Main results	16	(a) Give unadjusted estimates	(a – c): Main results		

For peer review only

		and, if applicable, confounder-adjusted estimates and their precision (e.g., 95% confidence interval). Make clear which confounders were adjusted for and why they were included<br>(b) Report category boundaries when continuous variables were categorized<br>(c) If relevant, consider translating estimates of relative risk into absolute risk for a meaningful time period	section		
Other analyses	17	Report other analyses done—e.g., analyses of subgroups and interactions, and sensitivity analyses	Not applicable		
**Discussion**					
Key results	18	Summarise key results with reference to study objectives	Page 11: lines 238 - 242		
Limitations	19	Discuss limitations of the study, taking into account sources of potential bias or imprecision. Discuss both direction and magnitude of any potential bias	Page 12: Lines 269 - 281	RECORD 19.1: Discuss the implications of using data that were not created or collected to answer the specific research question(s). Include discussion of misclassification bias, unmeasured confounding, missing data, and changing eligibility over time, as they pertain to the study being reported.	
Interpretation	20	Give a cautious overall interpretation of results considering objectives, limitations, multiplicity of analyses, results from similar studies, and other relevant evidence	Page 12: Lines 283 - 298		
Generalisability	21	Discuss the generalisability (external validity) of the study results	Page 14: lines 300 - 313		

For peer review only

**Other Information**					
Funding	22	Give the source of funding and the role of the funders for the present study and, if applicable, for the original study on which the present article is based	Page 15: lines 338		
Accessibility of protocol, raw data, and programming code		..		RECORD 22.1: Authors should provide information on how to access any supplemental information such as the study protocol, raw data, or programming code.	22.1

*Reference: Benchimol EI, Smeeth L, Guttmann A, Harron K, Moher D, Petersen I, Sørensen HT, von Elm E, Langan SM, the RECORD Working Committee. The REporting of studies Conducted using Observational Routinely-collected health Data (RECORD) Statement. *PLoS Medicine* 2015; in press.

*Checklist is protected under Creative Commons Attribution (CC BY) license.

For peer review only

Does attendance at a specialist antenatal clinic improve clinical outcomes in women with Class III obesity compared to standard care? : a retrospective case-note analysis.

Journal:	*BMJ Open*
Manuscript ID	bmjopen-2016-015218.R1
Article Type:	Research
Date Submitted by the Author:	23-Jan-2017
Complete List of Authors:	Denison, Fiona; The University of Edinburgh , MRC Centre for Reproductive Healht<br>MacGregor, Heather; University of Edinburgh Queen's Medical Research Institute<br>Stirrat, Laura; University of Edinburgh Queen's Medical Research Institute<br>Stevenson, Kerrie; University of Edinburgh Queen's Medical Research Institute<br>Norman, Jane; University of Edinburgh<br>Reynolds, Rebecca; University of Edinburgh,
**Primary Subject Heading**:	Obstetrics and gynaecology
Secondary Subject Heading:	Health services research
Keywords:	Obesity, Stillbirth, low birthweight

Specialist clinic improves outcomes in maternal obesity

Does attendance at a specialist antenatal clinic improve clinical outcomes in women with Class III obesity compared to standard care? a retrospective case-note analysis.

Denison FC*, MacGregor H, Stirrat LI, Stevenson, KJ, Norman JE, Reynolds RM

Denison FC: Reader and Honorary Consultant in Maternal and Fetal Health, Tommy's Centre for Maternal and Fetal Health, University of Edinburgh MRC Centre for Reproductive Health, Queen's Medical Research Institute, 47 Little France Crescent, Edinburgh, EH16 4TY

MacGregor H: Medical Student, University of Edinburgh

Stirrat LI: Clinical Research Fellow, Tommy's Centre for Maternal and Fetal Health, University of Edinburgh MRC Centre for Reproductive Health, Queen's Medical Research Institute, 47 Little France Crescent, Edinburgh, EH16 4TY

Stevenson, KJ: Medical Student, University of Edinburgh

Norman JE: Professor of Maternal and Fetal Health, Tommy's Centre for Maternal and Fetal Health, University of Edinburgh MRC Centre for Reproductive Health, Queen's Medical Research Institute, 47 Little France Crescent, Edinburgh, EH16 4TY

Reynolds RM: Professor of Metabolic Medicine, British Heart Foundation Centre for Cardiovascular Science, Queen's Medical Research Institute, 47 Little France Crescent, Edinburgh, EH16 4TY

Corresponding author:

Dr Fiona Denison

Tommy's Centre for Maternal and Fetal Health

For peer review only

MRC Centre for Reproductive Health

Queen's Medical Research Institute

Edinburgh

0131 242 6449

Fiona.Denison@ed.ac.uk

For peer review only

Abstract

Objectives: To determine whether attendance at a specialised multidisciplinary antenatal clinic for women with Class III obesity (BMI>40kg/m^2) is associated with improved clinical outcomes compared to standard antenatal care

Design: Retrospective cohort study using routinely collected data from electronic patient record

Setting: Community and hospital based antenatal care

Participants: Women with a singleton pregnancy with Class III obesity booked for antenatal care and delivered in one of two hospitals in NHS Lothian, Scotland, UK between 2008 – 2014. Maternal and offspring outcomes were compared in women who attended a specialised obesity clinic (n=511) compared to standard antenatal care (n=502).

Main Outcome Measures: Included stillbirth, low birthweight, gestational diabetes, induction of labour and caesarean section.

Results: Compared to standard care, women receiving specialist care were less likely to have a stillbirth (Odds Ratio (OR), 95% Confidence Interval (CI) 0.12, 0.06-0.97) and a low birthweight baby (OR, 95% CI 0.57, 0.33-0.99) and more likely to be screened for (100% vs 73.6%; <0.001) and diagnosed with (26.0% vs 12.5%; p<0.001) gestational diabetes, to require induction of labour (38.4% vs 29.9%, p=0.009), an elective (20.3% vs 17.7%; p<0.001) and emergency (23.9% vs 20.3%; p<0.001) caesarean section and attend antenatal triage one or more times during pregnancy (77.7% vs 53.1%; p<0.001). Women attending the specialist clinic had a higher BMI (44.5kg/m^2 (4.3) vs 43.2 kg/m^2 (3.1); p<0.001) and were more likely to be nulliparous (46.0% vs 24.9%; p<0.001). There were no other differences in maternal demographic or maternal and offspring outcomes between groups.

For peer review only

Conclusions: Attendance at a specialised antenatal clinic for obesity is associated with reduced rates of stillbirth and low birthweight and improved detection of gestational diabetes. The improvement in clinical outcomes is associated with an increase in healthcare attendance to obstetric triage and clinical interventions including induction of labour and caesarean section.

Keywords

Obesity, stillbirth, low birthweight

For peer review only

Specialist clinic improves outcomes in maternal obesity

Article Summary - Strengths and limitations of this study

- This study compares maternal and offspring outcomes in women with Class III obesity who attend a specialist obesity antenatal clinic compared to those who received standard care
- A strength of our study is that we were able to compare important clinical outcomes in women and offspring such as stillbirth and low birthweight.
- The use of routinely collected clinical data means that our results are relevant to clinical practice in which multiple different care pathways exist.
- The stillbirth findings and causality need to be interpreted with caution due to the small sample size and attenuation of findings in adjusted analyses.
- As a retrospective cohort study using routinely collected data from electronic patient record, results must be interpreted with caution because of potential bias from confounding factors.

For peer review only

Introduction

Maternal obesity is the most common co-morbidity of pregnancy. In the UK, approximately 20% of pregnant women are obese and 2% have very severe obesity (Class III obesity, body mass index BMI ≥ 40 kg/m^2) [1]. Maternal obesity is associated with increased risks for adverse maternal and offspring health including gestational diabetes mellitus (GDM), thromboembolic and hypertensive complications, caesarean section, macrosomia and stillbirth [2-5]. Managing these complications has significant cost implications for delivery of antenatal care [2, 4] [6].

There is recognition that obstetric management of the obese should be consultant led and involve a multi-disciplinary team to improve outcome [7, 8]. These recommendations are embedded in clinical guidelines and standards of care produced by a number of countries [8-13]. However, there is a paucity of evidence demonstrating that multidisciplinary care and adherence to guidelines results in improved maternal and offspring outcomes in maternal obesity. There is also less consensus about how multidisciplinary care should be delivered, and a concern that in areas of high obesity prevalence specialist obesity clinics are unlikely to be feasible due to cost and the numbers of women who would potentially need to be seen [13].

Women with Class III obesity are at particularly high risk of adverse maternal and offspring outcome [14]. In 2008 we therefore set up a specialist antenatal clinic for women with Class III obesity living in Edinburgh and the surrounding Lothian area with the aim of improving maternal and offspring outcomes. At their first antenatal appointment, which is generally prior to 12 weeks gestation, women with a BMI >40kg/m^2 are offered referral to the specialist clinic or can choose to continue to receive standard antenatal care. We have a pan-Lothian guideline for clinical management of pregnancies in women with obesity (Class I, II and III) so that the same care pathway is offered, regardless of who or where it is delivered. All women with Class III obesity should therefore receive the same standard of care. We hypothesised that maternal and offspring outcomes would be better in women who had their antenatal care provided by a multidisciplinary specialist clinic as opposed to receiving standard antenatal care. To test this hypothesis, we undertook a retrospective case-note review of all women with a BMI>40kg/m^2

For peer review only - http://bmjopen.bmj.com/site/about/guidelines.xhtml

who delivered in Lothian between 2008 and 2014 and compared clinical outcomes in women who attended for specialist antenatal care compared to those who received standard antenatal care.

Methods

Study population

We performed a retrospective case-note review of all women with Class III obesity with a singleton pregnancy who booked for antenatal care and delivered in either of two hospitals in NHS Lothian trust between 2008 and 2014. The Simpson Centre for Reproductive Health at the Royal Infirmary of Edinburgh is a tertiary referral centre with more than 6,500 deliveries per annum. St John's Hospital, Livingston, is a district general hospital with approximately 2,600 deliveries per annum. Women were excluded if they had not delivered by the end of December 2014, had a multiple pregnancy (n=28), or booked later than 20 weeks gestation (n=18) because this meant they would have missed the gestational window for early screening for GDM [15]. This study was an approved by our local audit committee.

Clinical care pathway

Women attending the specialist clinic at the Simpson Centre for Reproductive Health, Royal Infirmary of Edinburgh receive multidisciplinary consultant-led care throughout pregnancy from obstetricians, specialist midwives, diabetologists, anaesthetists, dieticians and other specialists as clinically indicated. At their first appointment (~10-16 weeks gestation), women are reviewed individually by a dietician with specialist expertise in weight management during pregnancy and given tailored advice about healthy eating and weight management during pregnancy. They are advised to have early screening for GDM with a fasting blood glucose between 12-16 weeks and late screening using a 75g oral glucose tolerance test between 24-28 weeks, as per the Scottish Intercollegiate Guidelines [15]. If a woman has pre-existing Type 2 diabetes or is diagnosed with GDM during pregnancy, her care remains within the specialist clinic. At each visit, women are weighed, counselled about the maternal and offspring risks associated

For peer review only - http://bmjopen.bmj.com/site/about/guidelines.xhtml

with maternal obesity, and their blood pressure is measured with appropriate sized cuffs. Women are commenced on 75mg aspirin if they have additional risk factors for pre-eclampsia such as a blood pressure of >140/90 mmHg at antenatal booking or primiparity as per national guidelines [16]. All women have postnatal thromboprophylaxis with low molecular weight heparin, with antenatal thromboprophylaxis being commenced if additional risk factors develop [16]. Fetal growth is monitored by serial growth scans at 28, 32 and 36 weeks. All women receive a personalised delivery plan and an anaesthetic review in the third trimester to discuss intrapartum pain management with specific consideration given to obesity related co-morbidities with implications for analgesia and anaesthesia.

Women who do not attend the specialist clinic receive guideline based consultant led care in hospital (tertiary or district general) or community based antenatal clinics. The main difference between specialist and standard care, is that if a woman receiving standard care develops a complication she needs to attend an additional separate specialist clinic, for example a diabetes clinic in the event she develops gestational diabetes. For women who attend the obesity clinic, this care is centralised in a single multi-professional clinic.

To compare maternal and offspring outcomes by antenatal care setting, women were categorised as 'Specialist care' if they attended for two or more appointments at the specialist clinic with the first appointment being before 20 weeks. The rationale for this was that such women would have received early dietary advice and counselling about the importance of attending for early screening for GDM. Women who did not attend the specialist clinic were categorised as receiving 'standard care'.

Data Collection

Maternal and offspring data were acquired from the maternity electronic patient records database TRAK (supplied by Intersystems), clinical biochemistry database APEX (ApexHealthware) and the neonatal unit electronic patient records database BadgerNet (supplied by Clevermed) systems.

For peer review only

The following data were collected from the maternal record at booking: maternal age, BMI (kg/m^2), ethnicity (white, other), parity (P0, P1, P2 or more), smoking status (current, former, never), deprivation quintile (a postcode based Scottish Index of Multiple Deprivation from 2012 with five groups ranging from most deprived index (1) to least deprived index (5))[17] and systolic and diastolic blood pressure (mmHg).

Maternal outcomes collected were hypertension (pre-existing, gestational, pre-eclampsia), diabetes (pre-existing, GDM), onset of labour (no labour, spontaneous onset, induced), delivery method (elective caesarean, emergency caesarean, instrumental, spontaneous vaginal), blood loss at delivery and antenatal obstetric triage attendances. The prevalence of GDM was determined according to (i) the rates of GDM from diagnoses entered into the electronic patient record and (ii) evaluating whether blood glucose values found on the electronic databases conferred a diagnosis of GDM. Diagnostic accuracy of GDM was determined according to Scottish Intercollegiate Guidelines which utilised the World Health Organisation (WHO) recommended thresholds [18] until March 2010 when updated thresholds were published based on the International Association of the Diabetes and Pregnancy Study Groups [19].

Offspring outcomes collected were gender, birthweight, birthweight centile [20], macrosomia (defined as a birthweight ≥4000g), low birthweight (defined as a birthweight ≤ 2500g), gestation of delivery, preterm birth (defined as birth <259 days gestation) and outcome (livebirth, stillbirth).

All data were anonymised with personal identifiers removed before analysis. To maximize accuracy and to minimise missing data all records were reviewed by HM and LS, glucose data was reviewed by KS and LS with any discrepancies reviewed by FD, RR. For stillbirths, a perinatal pathologist examined placental pathology as is routine clinical practice. HM and LS independently identified risk factors and categorised the likely causality of the stillbirths. Stillbirth causation was checked and verified by a third investigator (FD). All investigators were blinded to whether a woman received ‘specialist’ or ‘standard’ care until risk factors and likely causality was agreed for all stillbirths.

For peer review only - http://bmjopen.bmj.com/site/about/guidelines.xhtml

Statistical Analysis

Data were analysed using the Statistical Package for the Social Sciences (SPSS) Version 21. Differences in the characteristics and clinical outcomes between the women who attended the specialist obesity clinic and those who received standard care were tested using the student's t-test if the variable was continuous or the chi-squared test for categorical variables. Logistic regression was used to adjust for BMI and parity. A p-value <0.05 was considered statistically significant.

Results

Demographics

Maternal demographics are demonstrated in Table 1. Compared to standard care women who attended the specialist clinic had a higher BMI, and were more likely to be primiparous. There were no differences in age, ethnicity, smoking status, systolic or diastolic blood pressure at booking between attenders and non-attenders. There was a trend towards deprivation levels being different in those attending for specialist compared to standard care with more women from both the least and most deprived attending specialist care.

Maternal outcomes

Maternal outcomes are demonstrated in Table 2. After excluding women with pre-existing Type 1 and Type 2 diabetes, all women who attended the specialist clinic had a screening test with sufficient information being collected to confirm or exclude a diagnosis of GDM. In contrast, 26.4% (128/484) of those receiving standard care either had no screening test for GDM or insufficient information was collected for a diagnosis of GDM to be made. The clinical diagnosis of GDM from the patient record matched the diagnosis from blood glucose levels in all women who attended the specialist clinic. In contrast, in those receiving standard care, when the notes and actual blood glucose values were compared, the 'wrong' diagnosis was made in 17 women. One woman was incorrectly diagnosed with GDM when her screening test for GDM was normal. A further 16 woman had a positive diagnostic test for GDM

For peer review only - http://bmjopen.bmj.com/site/about/guidelines.xhtml

according to glucose values obtained during a glucose tolerance test but the diagnosis was missed and these women were incorrectly labelled as not having GDM (and did not therefore receive treatment).

Compared to those receiving standard care, women who attended the specialist clinic were more likely to have their labour induced, to have a caesarean or instrumental vaginal delivery. Specialist clinic attenders had a higher blood loss at delivery than those receiving standard care even after adjusting for mode of delivery, BMI, age and parity (p=0.02). They were also more likely to attend obstetric triage one or more times during pregnancy. Rates of pre-existing chronic hypertension and hypertensive complications (gestational hypertension and pre-eclampsia) were low in both attenders and non-attenders. Rates of Type 2 diabetes were higher in non-attenders compared to attenders.

Offspring outcomes

The clinical details for the offspring outcomes are demonstrated in Table 3. Compared to standard care, women attending for specialist care were less likely to have a stillbirth (Odds Ratio (OR), 95% Confidence Interval (CI) 0.12, 0.06-0.97) and a low birthweight baby (OR, 95% CI 0.57, 0.33-0.99). The lower stillbirth outcomes in women who attended for specialist care were attenuated in analyses adjusting for BMI and parity (adjusted OR (AOR), 95% CI 0.14, 0.02-1.17) but the lower risk of having a low birthweight baby was strengthened in adjusted analyses (AOR, 95% CI 0.52, 0.29-0.93). The clinical details of the women who had a stillbirth are demonstrated in Table 4. In women attending for standard care, an additional risk factor for stillbirth was identified in 7 women and a probable cause for stillbirth was identified in all 8 women. No additional risk factors or cause was identified in the one woman who had a stillbirth who attended the specialist clinic.

Discussion

In this retrospective case-note review, we demonstrated that women with Class III obesity who attended a specialist multidisciplinary antenatal clinic were less likely to have a stillbirth and low birthweight infant and more likely to be tested, correctly diagnosed with and treated for GDM, and to have an induction of

For peer review only - http://bmjopen.bmj.com/site/about/guidelines.xhtml

labour, caesarean section and higher blood loss at delivery compared to those receiving standard antenatal care. These differences in outcomes were accompanied by increased attendance at obstetric triage.

Main Findings

A key study finding was that rates of stillbirth and low birthweight were lower in women who attended the clinic compared to those who did. Compared to standard care, women who attended the specialist clinic had a higher BMI, and were more likely to be primiparous. Given that primiparity and higher BMI are independently associated with increased risk of stillbirth and low birthweight [21-23], we expected that rates of stillbirth and low birthweight would be higher in women receiving specialist as compared to standard care. However, we found the converse to be the case, with fewer stillborn and low birthweight babies being born to women attending the specialist clinic, even after adjusting for parity and BMI. We are uncertain why rates of low birthweight are lower in women attending the specialist clinic are lower since there are no differences in the length of gestation or frequency of preterm birth.

The stillbirth rate in women who attended the specialist clinic was 2 per 1000 compared to a rate of 7 per 1000 for women with a BMI>40kg/m^2 who delivered in Scotland in 2011 - 2012 [24]. To validate this finding, three investigators who were blinded to whether women received specialist or standard care independently checked the stillbirth data. It was striking that additional risk factors were identified in 7 and a cause for stillbirth identified in all 8 women who received standard care and who had a stillbirth but no additional risk factors or cause was identified in the one woman who had a stillbirth who attended the specialist clinic. We accept that rates of unexplained stillbirth are generally reported as being 20 – 25% which is much higher than what we found in our study. We therefore acknowledge the stillbirth findings and causality need to be interpreted with caution due to the small sample size and attenuation of findings in adjusted analyses. However it is tempting to speculate that the continuity of care together with the education of women by the multidisciplinary clinic team raised increased awareness of the importance of risk factors such as reduced fetal movements and this may have led to them presenting earlier to obstetric triage and being induced prior to stillbirth occurring. Future studies such as the AFFIRM clinical trial

For peer review only

(NCTT01777022, due to complete in 2017) are designed to address this in the general antenatal population.

Strengths and Limitations

A strength of our study is that we were able to compare important clinical outcomes in women and offspring such as stillbirth. We also used routinely collected clinical data meaning that our results are relevant to clinical practice in which multiple different care pathways exist. We accept that a limitation of our study is that this was a retrospective case-note review and our sample size was therefore limited by the study population. For the majority of data fields, other than smoking status (43.8% missing), there was a relatively low proportion of missing data. For the smoking variable, this was due to smoking status not being a mandatory field for recording on the electronic clinical record prior to 2012. The study was also not randomised, so women could choose whether to attend the specialist clinic. However, apart from differences in maternal BMI (albeit a small difference of uncertain clinical significance) and primiparity and a trend towards differences in deprivation status between women who attended for specialist compared to standard care, all other demographic factors were comparable between groups. Given that the clinical outcomes were better in women attending the specialist clinic who were arguably at higher risk than those attending standard care due to their higher BMI and more likely to be primiparous, we believe that our finding that multidisciplinary care improves clinical outcomes in pregnant women with Class III obesity compared to standard care is clinically important.

For peer review only

Interpretation

In other general and high-risk populations, pregnancy outcomes tend to be worse in women who either under- or do not attend for any antenatal care [25]. However, although we categorised women into women who attended for specialist and standard care this was only in relation to how their antenatal care was organised and not whether they did or did not attend for any antenatal care. In 1993, the landmark Changing Childbirth Report [26], which was built on the 1992 Winterton Report, reversed the official policy that hospital is always the safest place for birth and emphasised the importance of maternal choice,

For peer review only - http://bmjopen.bmj.com/site/about/guidelines.xhtml

control and continuity of carer for women. These recommendations, which were made over 20 years ago are still as relevant today, and frame the rhetoric and delivery of antenatal care across the UK [27-30]. In Lothian, all women receiving community-led care have a named midwife who coordinates their care. This midwife is part of a community team which has a defined case-load. This model ensures that there is continuity of care for a woman at both the individual midwife and midwifery team-level. If a woman is deemed high risk (such as would be the case in women with Class III obesity), she is also designated a named Consultant to oversee her care. Despite this model of continuity of care, our study demonstrates that maternal and offspring outcomes are better in women who attend a hospital based specialist clinic compared to those who receive standard antenatal care.

Although specialist clinics have been advocated as a way of improving maternal and offspring outcomes, there is currently a paucity of evidence from randomised controlled trials about the benefits and harms of specialist antenatal clinics compared with standard antenatal care for women [31]. For example, systematic reviews of randomised controlled trials have concluded that there is currently limited information to assess the role of specialist antenatal clinics for women with a multiple pregnancy [32] and no clear evidence that specialist clinics reduce the number of preterm births [33]. Given that the antenatal care pathway followed was the same in women who attended the specialist clinic and those who received standard care, it is not clear why maternal and offspring outcomes were better in women who attended the specialist clinic. A recent systematic review by Sandall et al. highlighted the importance of continuity of care, demonstrating that pregnant women receiving midwife-led continuity models of care had at least comparable clinical outcomes and were likely to experience less intervention [34]. It is therefore plausible that the continuity of care that the specialist multidisciplinary team provided enabled compromised pregnancies to be identified more accurately and interventions such as induction of labour to be targeted more appropriately compared to those women receiving standard care. It is also possible that staff providing standard antenatal care have less experience of Class III obesity and poorer access to appropriate facilities and equipment which may have adversely impacted their ability to provide optimal antenatal care to these high risk women.

For peer review only - http://bmjopen.bmj.com/site/about/guidelines.xhtml

Conclusion

In summary, our study demonstrates that attendance at a multidisciplinary specialist antenatal clinic improves maternal and offspring outcomes in women with Class III obesity. This challenges current recommendations that women with very Class III obesity can be effectively managed outside a specialist service. Further research is needed to identify the most appropriate and economic model of care for women with Class III obesity to optimise maternal and offspring outcomes.

Acknowledgements

We would like to acknowledge Mr Allyn Dick for assistance in extracting the clinical data from maternity TRAK.

Copyright

The Corresponding Author has the right to grant on behalf of all authors and does grant on behalf of all authors, a worldwide licence to the Publishers and its licensees in perpetuity, in all forms, formats and media (whether known now or created in the future), to i) publish, reproduce, distribute, display and store the Contribution, ii) translate the Contribution into other languages, create adaptations, reprints, include within collections and create summaries, extracts and/or, abstracts of the Contribution, iii) create any other derivative work(s) based on the Contribution, iv) to exploit all subsidiary rights in the Contribution, v) the inclusion of electronic links from the Contribution to third party material where-ever it may be located; and, vi) licence any third party to do any or all of the above.

Disclosure of Interests

The authors have no interests to disclose.

Contribution to authorship

For peer review only

FD and RR conceived the study and drafted the paper. FD, HM, LS, KS, JN and RR designed the study. FD, HM, LS, KS and RR acquired and analysed the data. All authors interpreted the data, revised the paper critically for important intellectual content and approved the final version.

Details of Ethics Approval

This study was an approved by our local audit committee.

Data Sharing

No additional data available

Funding

We acknowledge funding from Tommy's the Baby Charity. This work was carried out in the MRC Centre for Reproductive Health: Centre Grant MRC MR/N022556/1

For peer review only

Specialist clinic improves outcomes in maternal obesity

References

1. Report, C.f.M.a.C.E., *Maternal obesity in the UK: findings from a national project*, in *Centre for Maternal and Child Enquiries*2010: Chiltern Court, 188 Baker Street, London, NW1 5SD.
2. Denison, F.C., et al., *Association between maternal body mass index during pregnancy, short-term morbidity, and increased health service costs: a population-based study.* BJOG, 2014. **121**(1): p. 72-81; discussion 82.
3. Denison, F.C. and C. Chiswick, *Improving pregnancy outcome in obese women.* Proc Nutr Soc, 2011. **70**(4): p. 457-64.
4. Denison, F.C., et al., *Increased Maternal BMI is associated with an Increased Risk of Minor Complications during Pregnancy With Consequent Cost Implications.* BJOG, 2009. **116**(11): p. 1467-72.
5. Reynolds, R.M., et al., *Maternal and Offspring Outcomes of Pregnancies associated with Severe Obesity: Study design and preliminary data from the Hormones and Inflammation in Obese Pregnancy Study.* J of Developmental Origins of Health and Disease 2009: p. P7A-318.
6. Morgan, K.L., et al., *Obesity in pregnancy: a retrospective prevalence-based study on health service utilisation and costs on the NHS.* BMJ Open, 2014. **4**(2): p. e003983.
7. Ghaffari, N., S.K. Srinivas, and C.P. Durnwald, *The multidisciplinary approach to the care of the obese parturient.* Am J Obstet Gynecol, 2015. **213**(3): p. 318-25.
8. Jarvie, E. and J.E. Ramsay, *Obstetric management of obesity in pregnancy.* Semin Fetal Neonatal Med, 2010. **15**(2): p. 83-8.
9. *ACOG Committee Opinion No. 650: Physical Activity and Exercise During Pregnancy and the Postpartum Period.* Obstet Gynecol, 2015. **126**(6): p. e135-42.
10. *ACOG Practice Bulletin No 156: Obesity in Pregnancy.* Obstet Gynecol, 2015. **126**(6): p. e112-26.
11. Alavi, N., et al., *Comparison of national gestational weight gain guidelines and energy intake recommendations.* Obes Rev, 2013. **14**(1): p. 68-85.
12. Fitzsimons, K.J. and J. Modder, *Setting maternity care standards for women with obesity in pregnancy.* Semin Fetal Neonatal Med, 2010. **15**(2): p. 100-7.
13. RCOG, *Management of Women with Obesity in Pregnancy* in *RCOG* CMACE/RCOG, Editor 2010, CMACE/RCOG: London.
14. Lutsiv, O., et al., *The effects of morbid obesity on maternal and neonatal health outcomes: a systematic review and meta-analyses.* Obes Rev, 2015. **16**(7): p. 531-46.
15. Network, S.I.G., *Management of Diabetes: A national clinical guideline*, 2014, Scottish Intercollegiate Guidelines Network: Edinburgh.
16. NICE, *Hypertension in pregnancy: diagnosis and management: CG107*, 2011.
17. Government, T.S., *Scottish Index of Multiple Deprivation 2012. A National Statistics Publication for Scotland*, in *National Statistics*2012, The Scottish Government.
18. (SIGN), S.I.G.N., *Management of Diabetes. SIGN publication no.55*, 2001, SIGN: Edinburgh.
19. International Association of, D., et al., *International association of diabetes and pregnancy study groups recommendations on the diagnosis and classification of hyperglycemia in pregnancy.* Diabetes Care, 2010. **33**(3): p. 676-82.
20. Bonellie, S., et al., *Centile charts for birthweight for gestational age for Scottish singleton births.* BMC Pregnancy Childbirth, 2008. **8**: p. 5.
21. Aune, D., et al., *Maternal body mass index and the risk of fetal death, stillbirth, and infant death: a systematic review and meta-analysis.* JAMA, 2014. **311**(15): p. 1536-46.
22. Flenady, V., et al., *Stillbirths: the way forward in high-income countries.* Lancet, 2011. **377**(9778): p. 1703-17.
23. Shah, P.S. and L.B.W.P.T.b. Knowledge Synthesis Group on Determinants of, *Parity and low birth weight and preterm birth: a systematic review and meta-analyses.* Acta Obstet Gynecol Scand, 2010. **89**(7): p. 862-75.
24. ISD. *Stillbirth and Infant Deaths*. 2016 [cited 2016 14th November, 2016]; Available from: http://www.isdscotland.org/health-Topics/Maternity-and-Births/Stillbirth-and-Infant-Deaths/.

For peer review only

25. Raatikainen, K., N. Heiskanen, and S. Heinonen, *Under-attending free antenatal care is associated with adverse pregnancy outcomes.* BMC Public Health, 2007. **7**: p. 268.
26. Health, D.o., *Changing childbirth. Part 1: report of the Expert Maternity Group.*, D.o. Health, Editor 1993, Department of Health: London.
27. Health, D.o., *National Service Framework for children, young people and maternity services: maternity services*, 2004, Department of Health: London.
28. Health, D.o., *Matnerity matters: choice, access and continuity of care in a safe service*, 2007, Department of Health: London.
29. Government, S. *A Refreshed Framework for Maternity Care in Scotland*. 2011 [cited 2016 24th October, 2016]; Available from: http://www.gov.scot/resource/doc/337644/0110854.pdf.
30. *Better Births - Improving outcomes of maternity services in England*, N.M. Review, Editor 2016.
31. Dodd, J.M. and C.A. Crowther, *Specialised antenatal clinics for women with a multiple pregnancy for improving maternal and infant outcomes.* Cochrane Database Syst Rev, 2012(8): p. CD005300.
32. Dodd, J.M., T. Dowswell, and C.A. Crowther, *Specialised antenatal clinics for women with a multiple pregnancy for improving maternal and infant outcomes.* Cochrane Database Syst Rev, 2015(11): p. CD005300.
33. Whitworth, M., et al., *Specialised antenatal clinics for women with a pregnancy at high risk of preterm birth (excluding multiple pregnancy) to improve maternal and infant outcomes.* Cochrane Database Syst Rev, 2011(9): p. CD006760.
34. Sandall, J., et al., *Midwife-led continuity models versus other models of care for childbearing women.* Cochrane Database Syst Rev, 2016. **4**: p. CD004667.

For peer review only

Table 1: Demographics of population

	Specialist (n=511)	Standard (n=502)	P value
Age (years; mean (SD))	29.8 (5.4)	29.3 (5.5)	0.11
BMI (kg/m^2; mean (SD))	44.5 (4.3)	43.2 (3.1)	<0.001
Ethnicity (n (%))*			0.35
White	441 (94.6%)	432 (92.9%)	
Other	25 (5.4%)	33 (7.1%)	
Parity (n (%))			<0.001
0	235 (46.0%)	125 (24.9%)	
1	161 (31.5%)	212 (42.2%)	
2 or more	115 (22.5%)	165 (32.9%)	
Smoking status (n (%))*			0.51
Current	45 (17.2%)	42 (13.7%)	
Former	63 (24.0%)	79 (25.7%)	
Never	154 (58.8%)	186 (60.6%)	
Deprivation quintile (n (%))[1]*			0.07
1	140 (27.7%)	108 (22.2%)	
2	141 (27.9%)	150 (30.9%)	
3	95 (18.8%)	107 (22.0%)	
4	66 (13.1%)	74 (15.2%)	
5	63 (12.5%)	47 (9.7%)	
Systolic blood pressure (mmHg; mean (SD))	122 (11.9)	122 (11.1)	0.79
Diastolic blood pressure (mmHg)[1]	75 (9.0)	75 (8.0)	0.98

*missing data includes n=82 (8%) from ethnicity, n=444 (44%) from smoking and n=12 (1.2%) from deprivation quintile. Missing data is high from smoking as this was not a mandatory field on the electronic record until 2012; [1]deprivation quintile where 1 is the most and 5 the least deprived.

For peer review only

452 **Table 2: Maternal outcomes**

453

	Specialist N=511	Standard N=502	*P* value
Pre-existing co-morbidities			
Type 2 diabetes (n; %)	2 (0.4%)	12 (2.4%)	0.008
Hypertensive complications			0.27
Chronic hypertension (n; %)	16 (1.6%)	11 (1.1%)	
Gestational hypertension (n; %)	18 (1.8%)	16 (1.6%)	
Pre-eclampsia (n; %)	31 (3.1%)	25 (2.5%)	
Gestational diabetes*			
Screening/diagnostic test performed (n; %)	496 (100%)	356 (73.6%)	<0.001
Prevalence (n; %)	129 (26.0%)	61 (12.5%)	<0.001
Labour and delivery			
Onset labour (n; %)			0.009
No labour	111 (21.7%)	109 (21.7%)	
Spontaneous onset	204 (39.9%)	243 (48.4%)	
Induction	196 (38.4%)	150 (29.9%)	
Delivery method (n; %)			
Elective caesarean	103 (20.2%)	89 (17.7%)	<0.001
Emergency caesarean	122 (23.9%)	102 (20.3%)	
Instrumental	56 (11.0%)	23 (4.6%)	
Spontaneous vertex	229 (44.9%)	288 (57.4%)	
Blood loss at delivery (mls; mean (SD))	575 (464)	465 (387)	<0.001
Obstetric triage attendances (n; %)			<0.001
0	108 (21.1%)	229 (45.6%)	
1	132 (25.8%)	104 (20.7%)	
2	93 (18.2%)	70 (13.9%)	
3 or more	172 (33.7%)	93 (18.5%)	

454 *Denominator excludes women with pre-existing diabetes (type 1 or 2) or those who were not managed at the
455 tertiary referral centre. In women who attended for specialist and standard care, the prevalence is based on blood
456 glucose levels and not the clinical diagnosis recorded in the notes.

457

458

459

460

461

For peer review only

For peer review only - http://bmjopen.bmj.com/site/about/guidelines.xhtml

Table 3: Offspring outcomes

	Specialist N=511	Standard N=502	Significance (*P* value)
Gender (n; %)			0.34
Female	238 (46.6%)	249 (49.6%)	
Male	273 (53.4%)	253 (50.4%)	
Birthweight (g; mean (SD))	3576 (635)	3559 (664)	0.69
Macrosomia[1] (n; %)	31 (6.1%)	26 (5.2%)	0.54
Low birthweight[2] (n; %)	21 (4.1%)	35 (7.0%)	0.04
Gestation (days; mean (SD))	277 (14.1)	277 (14.7)	0.82
Preterm birth[3] (n; %)	40 (7.8%)	39 (8.4%)	0.97
Outcome (n; %)			
Livebirth	510 (99.8%)	494 (98.4%)	0.02
Stillbirth	1 (0.2%)	8 (1.6%)	

[1]macrosomia defined as birthweight of 4000g or more; [2]low birthweight defined as birthweight of 2500g or lower; [3]preterm birth defined as birth before 259 days gestation.

For peer review only

Table 4: Details of stillbirths

Case	Demographics: Age (years)	Parity	BMI (kg/m^2)	Risk factors	Outcome	Birthweight centile[1]	Cause
ST1	31	P2	42	Smoker, type 2 diabetes, RFM	33+5 weeks, male, 2050g	25th – 50th	Uncontrolled hypertension, abruption
ST2	32	P1	42	No risk factors	30+5 weeks, female, 700g,	<3rd	IUGR, placental insufficiency
ST3	38	P4	42	RFM	37 weeks, male, 2720g,	10th – 25th	Severe pre-eclampsia, abruption
ST4	32	P2	45	Smoker, RFM	36 weeks, male, 2160g,	5th – 10th	Acute intra-uterine hypoxia
ST5	26	P2	47	Smoker, RFM, isolated congenital anomaly	35+5 weeks, female, 2155g,	10th – 25th	Congenital anomaly
ST6	32	P2	52	Smoker	30+5 weeks, female, 1620g,	75th – 90th	Abruption
ST7	27	P2	40	Type 2 diabetes, RFM	38+2 weeks, male, 3370g,	50th – 75th	Poorly controlled diabetes
ST8	21	P0	40	Smoker	26+3 weeks, female, 750g,	25th – 50th	IUGR, placental insufficiency
SD1	20	P1	41	No risk factors	39+5 weeks, male, 3725g,	50th – 75th	Unexplained

Key: ST specialist, S standard, [1] birthweight centile defined by (Bonellie et al., 2008); BMI body mass index, RFM reduced fetal movements, IUGR intrauterine growth restriction, placental insufficiency diagnosed by perinatal pathologist

For peer review only

The RECORD statement – checklist of items, extended from the STROBE statement, that should be reported in observational studies using routinely collected health data.

	Item No.	STROBE items	Location in manuscript where items are reported	RECORD items	Location in manuscript where items are reported
**Title and abstract**					
	1	(a) Indicate the study's design with a commonly used term in the title or the abstract (b) Provide in the abstract an informative and balanced summary of what was done and what was found	(a) Page 1<br>(b) Page 4-5	RECORD 1.1: The type of data used should be specified in the title or abstract. When possible, the name of the databases used should be included.<br><br>RECORD 1.2: If applicable, the geographic region and timeframe within which the study took place should be reported in the title or abstract.<br><br>RECORD 1.3: If linkage between databases was conducted for the study, this should be clearly stated in the title or abstract.	1.1: Lines 52-53<br><br>1.2: Lines 57-58<br><br>1.3: Not applicable
**Introduction**					
Background rationale	2	Explain the scientific background and rationale for the investigation being reported	Page 6 &7, lines 83 - 111		
Objectives	3	State specific objectives, including any prespecified hypotheses	Page 6 & 7, lines 106 - 111		
**Methods**					
Study Design	4	Present key elements of study design early in the paper	Page 7 & 8, lines 117 - 155		
Setting	5	Describe the setting, locations, and relevant dates, including periods of recruitment, exposure, follow-up, and data collection	Page 7, lines 117 - 124		
Participants	6	*(a) Cohort study* - Give the eligibility criteria, and the	(a) Page 7 & 8, lines 127 – 155	RECORD 6.1: The methods of study population selection (such as codes or	6.1: Lines 152 – 155

For peer review only

		sources and methods of selection of participants. Describe methods of follow-up<br>*Case-control study* - Give the eligibility criteria, and the sources and methods of case ascertainment and control selection. Give the rationale for the choice of cases and controls<br>*Cross-sectional study* - Give the eligibility criteria, and the sources and methods of selection of participants<br><br>*(b) Cohort study* - For matched studies, give matching criteria and number of exposed and unexposed<br>*Case-control study* - For matched studies, give matching criteria and the number of controls per case	(b) Page 8, lines 152 - 155	algorithms used to identify subjects) should be listed in detail. If this is not possible, an explanation should be provided.<br><br>RECORD 6.2: Any validation studies of the codes or algorithms used to select the population should be referenced. If validation was conducted for this study and not published elsewhere, detailed methods and results should be provided.<br><br>RECORD 6.3: If the study involved linkage of databases, consider use of a flow diagram or other graphical display to demonstrate the data linkage process, including the number of individuals with linked data at each stage.	6.2: Not applicable<br><br>6.3: Not applicable
Variables	7	Clearly define all outcomes, exposures, predictors, potential confounders, and effect modifiers. Give diagnostic criteria, if applicable.	Page 8 & 9: lines 158 - 189	RECORD 7.1: A complete list of codes and algorithms used to classify exposures, outcomes, confounders, and effect modifiers should be provided. If these cannot be reported, an explanation should be provided.	Page 8 & 9: lines 158 - 189
Data sources/ measurement	8	For each variable of interest, give sources of data and details of methods of assessment (measurement).<br>Describe comparability of assessment methods if there is more than one group	Page 8 & 9: lines 158 - 189		
Bias	9	Describe any efforts to address potential sources of bias	Page 9: lines 184 - 189		
Study size	10	Explain how the study size was	Page 7: lines 117 -		

For peer review only

		arrived at	121		
Quantitative variables	11	Explain how quantitative variables were handled in the analyses. If applicable, describe which groupings were chosen, and why	Page 8 & 9: lines 158 - 189		
Statistical methods	12	(a) Describe all statistical methods, including those used to control for confounding<br>(b) Describe any methods used to examine subgroups and interactions<br>(c) Explain how missing data were addressed<br>(d) *Cohort study* - If applicable, explain how loss to follow-up was addressed<br>*Case-control study* - If applicable, explain how matching of cases and controls was addressed<br>*Cross-sectional study* - If applicable, describe analytical methods taking account of sampling strategy<br>(e) Describe any sensitivity analyses	(a) – (e) Page 10: lines 191 - 196		
Data access and cleaning methods		..		RECORD 12.1: Authors should describe the extent to which the investigators had access to the database population used to create the study population.<br><br>RECORD 12.2: Authors should provide information on the data cleaning methods used in the study.	12.1: Page 9, lines 184 – 189<br><br>12.2: Page 9, lines 184 - 189
Linkage		..		RECORD 12.3: State whether the study included person-level, institutional-	12.3: Not applicable

For peer review only

				level, or other data linkage across two or more databases. The methods of linkage and methods of linkage quality evaluation should be provided.	
**Results**					
Participants	13	(a) Report the numbers of individuals at each stage of the study (*e.g.*, numbers potentially eligible, examined for eligibility, confirmed eligible, included in the study, completing follow-up, and analysed)<br>(b) Give reasons for non-participation at each stage.<br>(c) Consider use of a flow diagram	(a - b) Page 6: lines 121 – 124 and page 10: lines 200<br>(c) Not applicable	RECORD 13.1: Describe in detail the selection of the persons included in the study (*i.e.,* study population selection) including filtering based on data quality, data availability and linkage. The selection of included persons can be described in the text and/or by means of the study flow diagram.	13.1: Page 6: lines 121 – 124 and page 10: lines 200
Descriptive data	14	(a) Give characteristics of study participants (*e.g.*, demographic, clinical, social) and information on exposures and potential confounders<br>(b) Indicate the number of participants with missing data for each variable of interest<br>(c) *Cohort study* - summarise follow-up time (*e.g.*, average and total amount)	(a) Page 9: lines 199 – 203<br><br>(b) Table 1<br><br>(c) Not applicable		
Outcome data	15	*Cohort study* - Report numbers of outcome events or summary measures over time<br>*Case-control study* - Report numbers in each exposure category, or summary measures of exposure<br>*Cross-sectional study* - Report numbers of outcome events or summary measures	Page 9 & 10, lines 205 – 235 and Tables 1 - 4		
Main results	16	(a) Give unadjusted estimates	(a – c): Main results		

For peer review only

		and, if applicable, confounder-adjusted estimates and their precision (e.g., 95% confidence interval). Make clear which confounders were adjusted for and why they were included<br>(b) Report category boundaries when continuous variables were categorized<br>(c) If relevant, consider translating estimates of relative risk into absolute risk for a meaningful time period	section		
Other analyses	17	Report other analyses done—e.g., analyses of subgroups and interactions, and sensitivity analyses	Not applicable		
**Discussion**					
Key results	18	Summarise key results with reference to study objectives	Page 11: lines 238 - 242		
Limitations	19	Discuss limitations of the study, taking into account sources of potential bias or imprecision. Discuss both direction and magnitude of any potential bias	Page 12: Lines 269 - 281	RECORD 19.1: Discuss the implications of using data that were not created or collected to answer the specific research question(s). Include discussion of misclassification bias, unmeasured confounding, missing data, and changing eligibility over time, as they pertain to the study being reported.	
Interpretation	20	Give a cautious overall interpretation of results considering objectives, limitations, multiplicity of analyses, results from similar studies, and other relevant evidence	Page 12: Lines 283 - 298		
Generalisability	21	Discuss the generalisability (external validity) of the study results	Page 14: lines 300 - 313		

Other Information					
Funding	22	Give the source of funding and the role of the funders for the present study and, if applicable, for the original study on which the present article is based	Page 15: lines 338		
Accessibility of protocol, raw data, and programming code		..		RECORD 22.1: Authors should provide information on how to access any supplemental information such as the study protocol, raw data, or programming code.	22.1

*Reference: Benchimol EI, Smeeth L, Guttmann A, Harron K, Moher D, Petersen I, Sørensen HT, von Elm E, Langan SM, the RECORD Working Committee. The REporting of studies Conducted using Observational Routinely-collected health Data (RECORD) Statement. *PLoS Medicine* 2015; in press.

*Checklist is protected under Creative Commons Attribution (CC BY) license.

For peer review only
